# Identification of a functional circRNA-miRNA-mRNA regulatory network in infantile hemangioma by bioinformatics analysis

**DOI:** 10.1097/MD.0000000000030791

**Published:** 2022-09-30

**Authors:** Da Gu, Huanmin Lou, Yang Li, Guangqi Xu

**Affiliations:** a Department of Plastic Surgery, Central Hospital Affiliated to Shandong First Medical University, Jinan, Shandong, China; b Department of Burn and Plastic Surgery, Shandong Provincial Hospital Affiliated to Shandong First Medical University, Jinan, Shandong, China.

**Keywords:** bioinformatics, ceRNA, circRNA, hub gene, infantile hemangioma

## Abstract

Several circRNA have been reported to serve critical roles in various biological processes of human body. The present study aimed to build a circRNA-based competing endogenous RNA (ceRNA) network and explore the regulatory mechanisms of circRNA in infantile hemangiomas (IH). Differentially expressed circRNA, miRNA, and mRNA were downloaded from the gene expression synthesis (GEO) microarray database (GSE98795, GSE69136, and GSE127487). Cancer-specific circRNA database (CSCD), miRDB and Targetscan were employed to predict the targets of RNA. A total of 855 DEcircRNAs, 69 differentially expressed miRNAs (DEmiRNAs), and 3233 differentially expressed mRNAs (DEmRNAs) appeared as genes that were aberrantly expressed in IH. The circRNA-miRNA-mRNA network was constructed based on 108 circRNAs, 7 miRNAs, 274 mRNAs in IH. Gene ontology (GO) and Kyoto encyclopedia of genes and genomes (KEGG) pathway analysis indicated hypoxia-inducible factors (HIF)-1 signaling pathway and Notch signaling pathway were significantly enriched in IH with being constructed a ceRNA regulatory network. Furthermore, protein-protein interaction (PPI) network and Cytoscape showed the top 10 hub genes that regulate angiogenesis, namely FBXW7, CBLB, HECW2, FBXO32, FBXL7, KLHL5, EP300, MAPK1, MEF2C, and PLCG1. Our findings provide a deeper understanding the circRNA-related ceRNA regulatory mechanism in IH. This study further perfected the circRNA-miRNA-mRNA regulatory network related to IH and explored the potential function of mRNA in this network. It provides more understanding for the circRNA-related ceRNA regulation mechanism in the pathogenesis of IH.

## 1. Introduction

Infant hemangioma is a common vascular tumor that occurs during infancy and has a higher incidence in females and preterm babies, where its incidence rises by 3% to 10%.^[1]^ Most IHs possess a unique life cycle composed of proliferation and involution stage. IHs spread rapidly in the first year, but deep hemangiomas tend to grow later and longer than superficial hemangiomas. The proliferation stage of IH has different durations, sometimes up to 12 or 14 months, or even up to 2 years, and then spontaneously subsides until 3 to 7 years old.^[2–4]^ Rapid tumor growth in the proliferative stage may cause complications like ulcers, scars, disfigurement and may even become life-threatening.^[5]^

Hypoxia has been associated with IH pathogenesis, Hypoxia-inducible factors (HIF) pathway and renin-angiotensin system (RAS).^[4]^ Hypoxia is triggered and maintained by maternal hypoxia stress and the infant’s hypoxia-inducing factors. Glucose transporter 1 (GLUT-1) has been identified as a highly selective marker of IH.^[6–8]^ GLUT-1, vascular endothelial growth factor A (VEGF-A) and insulin-like growth factor 2 are HIF-1α targets. RAS may influence IH endothelial cell proliferation. IH endothelial cells in the proliferative phase express angiotensin-converting enzyme and angiotensin receptor II, which are RAS components. High renin levels and local expression of angiotensin-converting enzyme in the serum is hypothesized to elevate angiotensin II levels, which together with VEGF drives cell proliferation. This theory may explain the mechanism underlying beta blockers.^[9]^ However, IH pathogenesis remains unknown.

Majority of the human genome is transcribed into non-coding RNA and most of them can not be encoded as proteins.^[10]^ CircRNAs are a class of non-coding RNAs derived from precursor mRNA. Unlike linear RNAs, circRNAs form covalent-closed continuous loops without 5’ to 3’ polarities and poly (A) tails. This structure confers stability to circRNA and they are not easily degraded by RNase R.^[11]^ CircRNAs are reported to modulate various processes, including the occurrence, metastasis and cancer resistance to therapy.^[12]^ CircRNA acts transcriptionally and post-transcriptionally through various functional mechanisms, including acting as RNA binding protein sponges and protein scaffolds,^[13]^ translate proteins,^[14]^ RNAP II elongation,^[15]^ RNA-RNA interactions,^[15]^ and RNA maturation.^[16]^ CircRNA sponge miRNA, reducing their cytoplasmic availability, thereby relieving miRNA-suppressed gene expression. This is also a mechanism by which circRNA works. CircAP2A2 has been reported to promote IH proliferation and invasion by regulating the miR-382-5p/VEGFA axis.^[17]^ In addition, the study identified 249 differentially expressed candidate genes in the IH group.^[18]^ Therefore, circRNAs play an important role in the proliferation and invasion of infantile hemangiomas (IH). Currently, circRNA mechanisms of action in IH are poorly understood.

In this study, we constructed a regulatory network consisting of 108 circRNAs, 7 miRNAs, and 274 mRNAs through multiple gene expression synthesis (GEO) databases and some online prediction sites. The gene ontology (GO) term, Kyoto encyclopedia of genes and genomes (KEGG), and protein-protein interaction (PPI) network analyses were done to understand the potential role of circRNAs in IH pathogenesis. This study uses the ceRNA network to explain the pathogenesis of IH and provide basic theoretical research for the treatment of IH

## 2. Materials and Methods

### 2.1. Download datasets

Microarray data was obtained from GEO (http://www.ncbi.nlm.nih.gov/gds/), and the inclusion criteria were infants younger than 24 months who had not receive treatment for IH. The mRNA dataset from GSE127487 includes 23 hemangioma tissues and 5 skin tissues, among which 12 hemangioma tissues and 5 normal skin tissue meet the inclusion criteria. The miRNA dataset, GSE69136, includes 12 hemangiomas, 8 lymphatic malformations, and 4 skin tissues, of which 6 hemangiomas and 3 normal skin tissue meet the inclusion criteria. The circRNA dataset from GSE98795 includes 4 hemangioma tissues and 4 matched non-tumor tissues, all meet the inclusion criteria. Use the R package “GEOquery” was used to download the expression matrix, platform information and clinical information. A total of 34 samples were included in this study, including 22 hemangioma samples and 12 normal samples.

### 2.2. Differential expression analysis of circRNA, miRNA, and mRNA

The R package “GEOquery” was used to download the expression matrix, platform information and clinical information. To further control for heterogeneity, we preprocessed the data before performing the difference analysis, including retaining the probe with the largest mean as the gene expression value if multiple probes correspond to a gene, data correction between groups, and log transformation. Differentially expressed RNA was analyzed using the limma package based on the Bioconductor software package. The criteria for selecting DEcircRNA, DEmiRNA and DEmRNA were *P* value = <.05 and | log2FC |> 1.

### 2.3. Construction of circRNA-miRNA-mRNA regulatory network

Based on the results of differential expression analysis, the cancer-specific circRNA (CSCD) (http://gb.whu.edu.cn/CSCD/) was used to predict the interaction of DEcircRNAs and DEmiRNAs. Based on the differentially expressed circRNAs and miRNAs, we initially screened the circRNA-miRNA axis. DEmiRNAs included in the circRNA-miRNA axis are called intersection DEmiRNAs (IDEmiRNAs), and DEcircRNAs are called intersection DEcircRNAs (IDEcircRNAs).

To predict the interaction between IDEmiRNA and DEmRNA, we used Targetscan (http://www.targetscan.org/vert_72/) and miRDB (http://www.mirdb.org/) online prediction website. We downloaded the prediction target file (Summary_Counts.default_predictions.txt) that predicted all miRNAs from the Targetscan online prediction website. The prediction target file (miRDB_v6.0_prediction_result.txt) that predicts all miRNAs was downloaded from miRDB online prediction website. The interaction between IDEmiRNA and DEmRNA was jointly predicted using the above 2 databases. The ceRNA network was further constructed using the shared miRNA-mRNA axis screened by the 2 databases. In the shared miRNA-mRNA axis, the IDEmiRNAs were called final DEmiRNA(FDEmiRNA) and the DEmRNAs were called final DEmRNA(FDEmRNA). The IDEcircRNA-IDEmiRNA axis containing FDEmiRNA was called the final circRNA-miRNA axis, and the circRNA on the axis was called the final IDEcircRNA (FDEcircRNA). Thus, the circRNA-miRNA-mRNA regulatory network was constructed based on FDEcircRNA, FDEmiRNA, and FDEmRNA.

### 2.4. Functional enrichment analysis

The R package, “clusterProfiler” was used to perform FDEmRNA KEGG analysis in the ceRNA network,^[19–21]^ while the R package, “pathview,” was used to draws a signal path diagram. *P* = <.05 was set as cutoff threshold.

### 2.5. Construction of PPI network and hub gene analysis

Based on the identified FDEmRNA, the search tool for the retrieval of interaction genes (STRING, https://string-db.org/) used to construct a PPI network.^[22]^ First, we mapped FDEmRNA to STRING to assess PPI information, with a confidence score > 0.4 as cutoff. The PPI network was then visualized using Cytoscape software.^[23]^ At last, key genes in PPIs were selected using maximum clique centrality in the CytoHubba plugin.

### 2.6. Statistical analysis

Statistical analyses were done using the R software (version 3.6.1; https://www.R-project.org), Statistical significance was set at *P* < .05.

## 3. Results

### 3.1. Identification of DEmRNA, DEmiRNA, DEcircRNA

**Figure 1.**
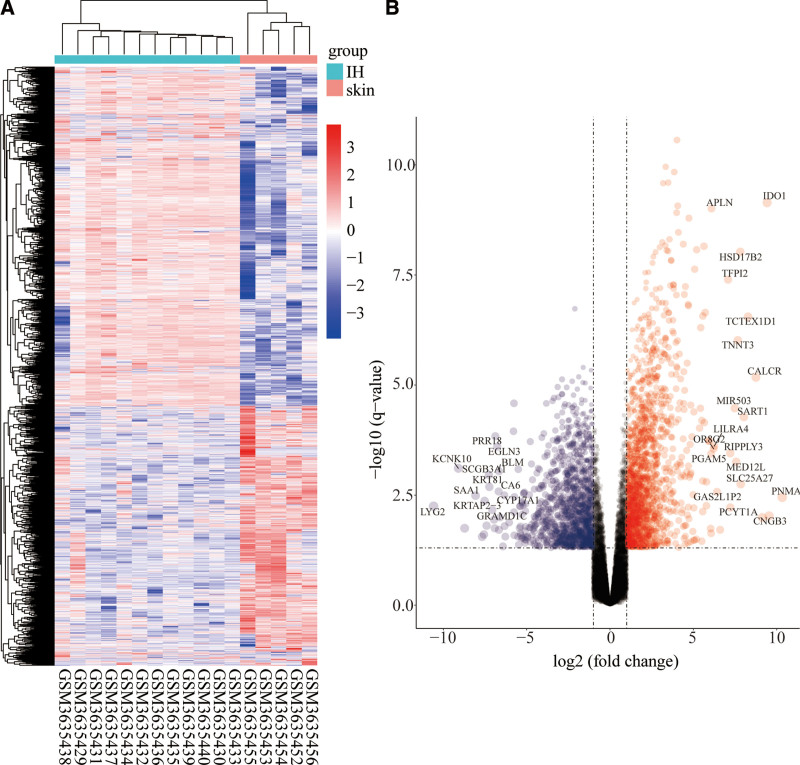
Difference analysis based on mRNA dataset GSE127487. (A) Heatmap shows the expression profiles of different genes in different groups. (B) The volcano map shows the distribution of different genes in different groups.

**Figure 2.**
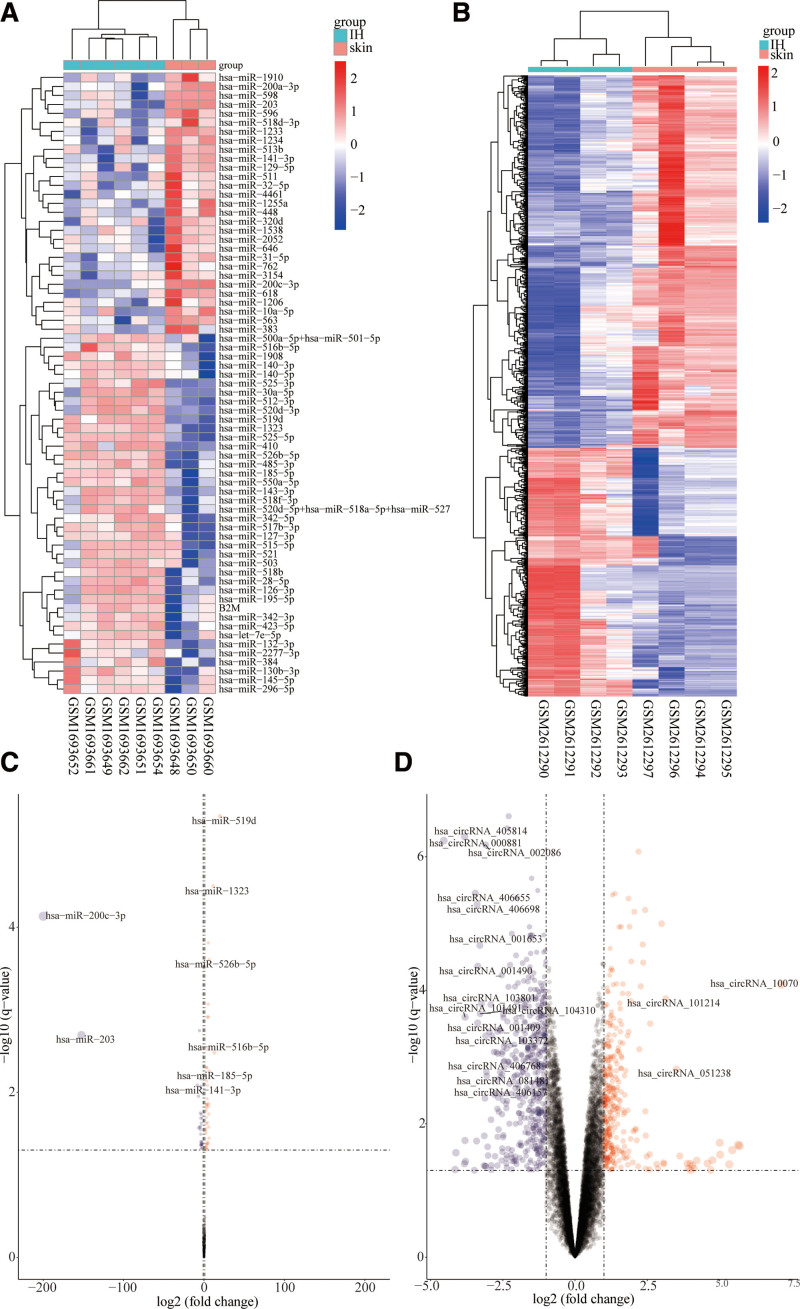
Difference analysis based on miRNA dataset GSE69136 and circRNA dataset GSE98795. (A) Heatmap shows the expression profile of differential genes in miRNA dataset GSE69136. (B) volcano map shows the distribution of differential genes in miRNA dataset GSE69136. (C) Heatmap shows the expression profile of differential genes in circRNA dataset GSE98795. (D) Volcano graph shows distribution of differential genes in the circRNA dataset GSE98795.

GSE127487, GSE69136 and GSE98795 datasets were downloaded from GEO. Basic information about the 3 datasets is shown in Table 1. The R package, limma, was used to conduct differential mRNA analysis on dataset GSE127487 and revealed 3233 DEmRNAs. Of these, 1854 were upregulated and 1379 downregulated (Fig. 1). Differential analysis of the miRNA dataset GSE69136 using the R package limma identified 69 DEmiRNA, of which 40 were upregulated and 29 downregulated (Fig. 2A and B). The differential analysis of the circRNA dataset GSE98795 identified 855 DEcircRNA (Fig. 2C and D). In view of the fact that the authors who used dataset GSE98795 had verified differential expression of some circRNAs in IH using PCR, 5 circRNAs (has_circRNA_102116, has_circRNA_051239, has_circRNA_102039, has_circRNA_023016, and has_circRNA_001654) identified as differentially expressed were excluded from subsequent analysis. Of the identified differentially expressed circRNAs, 343 were upregulated and 512 downregulated.

**Table 1:** Basic information of the 3 microarray datasets from GEO.

Datasets		GSE98795	GSE69136	GSE127487
Type		circRNA	miRNA	mRNA
Platform		GPL21825	GPL19765	GPL10558
Tissue	IH	4	6	12
	Skin	4	3	5
Age (mo)	IH	5.00 ± 1.41	12.32 ± 5.32	9.00 ± 3.13
	Skin	5.00 ± 1.41	15.67 ± 1.15	NA

GEO = gene expression synthesis.

### 3.2. Searching for the relationship among circRNA, miRNA, and mRNA

**Figure 3.**
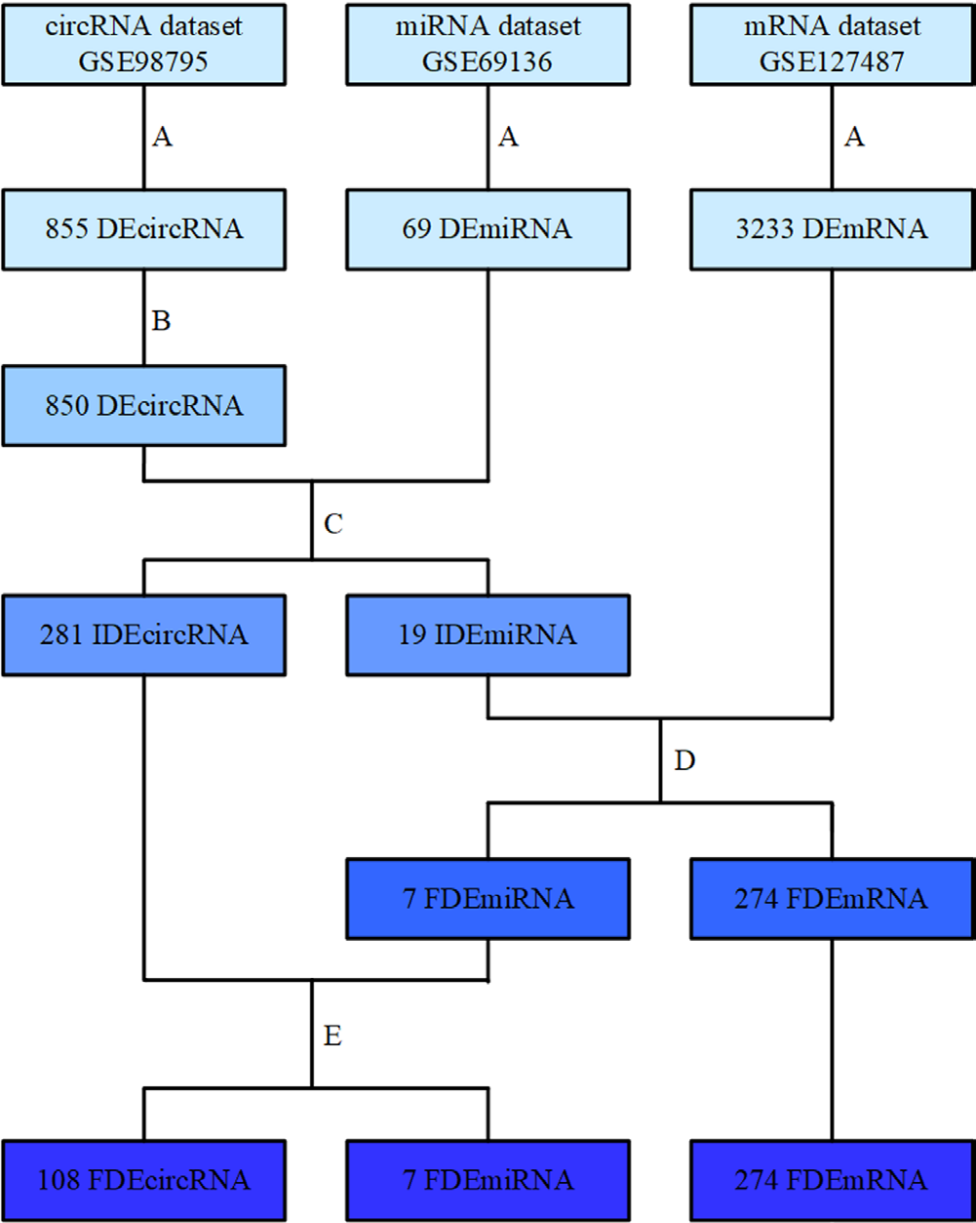
Flow chart of circRNA-miRNA-mRNA network construction. (A) “limma” R package was used for difference analysis, (B) remove 5 circRNAs without differences, (C) circRNA-miRNA axis construction using CSCD database, (D) the constructed miRNA-mRNA axis based on miRDB and Targetscan, (E) the constructed network based on circRNA-miRNA axis and miRNA-mRNA axis.

Using a variety of online prediction tools, 3233 DEmRNA, 69 DEmiRNA and 855 DEcircRNA were used to predict target genes (Fig. 3). First, CSCD website was used to predict the interaction of 850 DEcircRNAs and 69 DEmiRNAs. We initially identified 373 circRNA-miRNA axes, including 281 IDEcircRNA and 19 IDEmiRNA.

Next, miRDB and Targetscan databases were used to predict the interaction of 19 IDEmiRNAs and 3233 DEmRNAs This analysis identified 315 miRNA-mRNA axes, including 7 FDEmiRNA and 274 FDEmRNA.

Among 373 circRNA-miRNA axes that were initially screened, we removed axes that did not contain FDEmiRNAs. Finally, 108 FDEcircRNAs corresponding to 7 FDEmiRNAs was screened again and named FDEcircRNA. Based on 5403 circRNA-miRNA-mRNA axis, we constructed a ceRNA network for IHs, including 274 FDEmRNAs, 7 FDEmiRNAs and 108 FDEcircRNAs.

### 3.3. Construction of circRNA-miRNA-mRNA network

**Figure 4.**
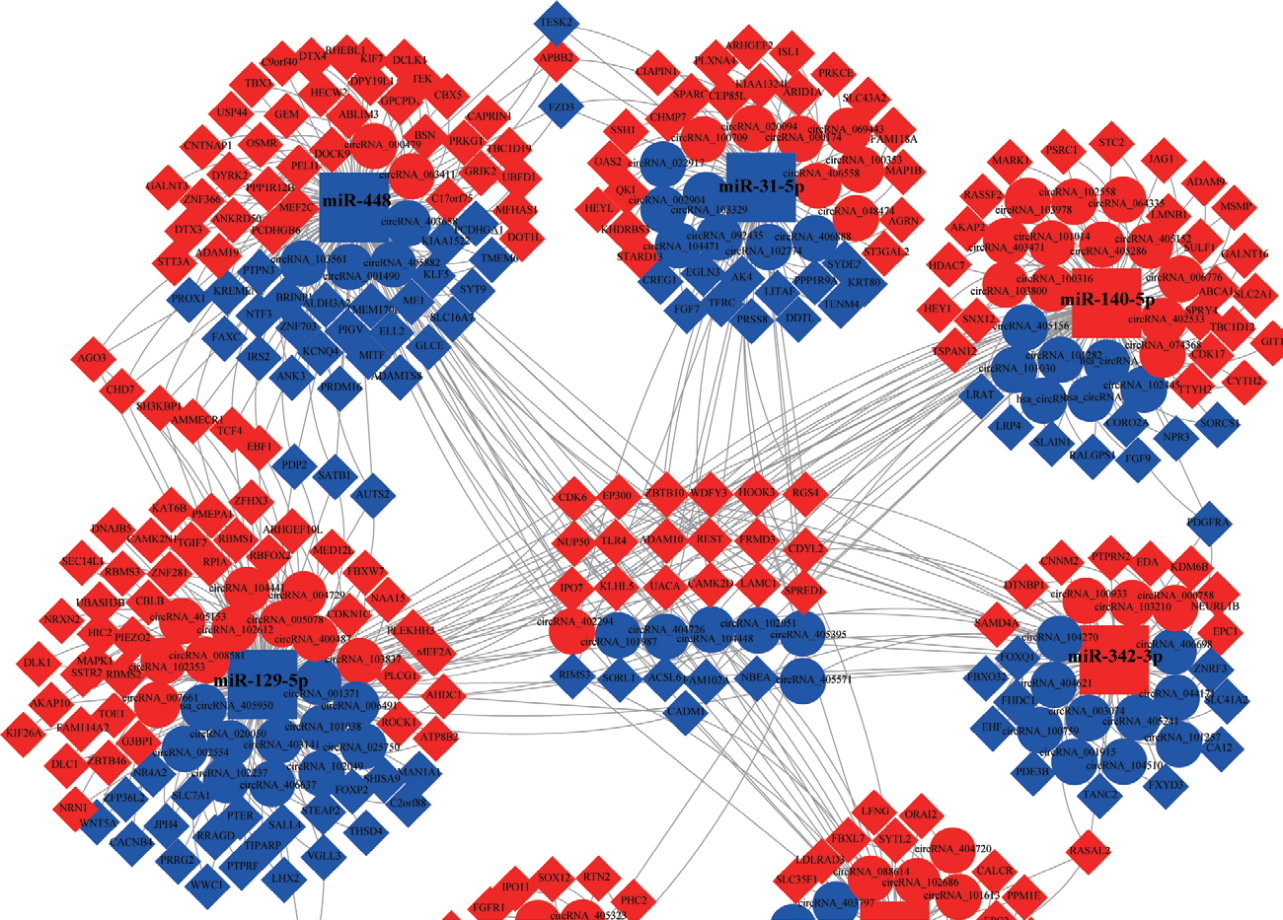
Visualization of circRNA-miRNA-mRNA regulatory network. Circle, diamond and square represent circRNA, miRNA and mRNA, respectively. Red represents upregulated RNA and blue represents downregulated RNA.

From the previous data analysis, our ceRNA network has 5403 circRNA-miRNA-mRNA axes, including 108 circRNA (50 upregulated and 70 downregulated), and 7 miRNAs (4 upregulated and 3 downregulated) and 274 mRNA (174 upregulated and 100 downregulated) (see Table S1–S4, http://links.lww.com/MD/H409; http://links.lww.com/MD/H410;http://links.lww.com/MD/H411; http://links.lww.com/MD/H412, Supplemental Content, which illustrates the ceRNA axis and differentially expressed circRNAs, miRNAs and mRNAs in the axis). Seven miRNAs had 315 targeting relationships with 274 mRNAs, and 7 miRNAs had 117 targeting relationships with 108 circRNAs (Fig. 4).

### 3.4. GO and KEGG analysis of FDEmRNA

**Figure 5.**
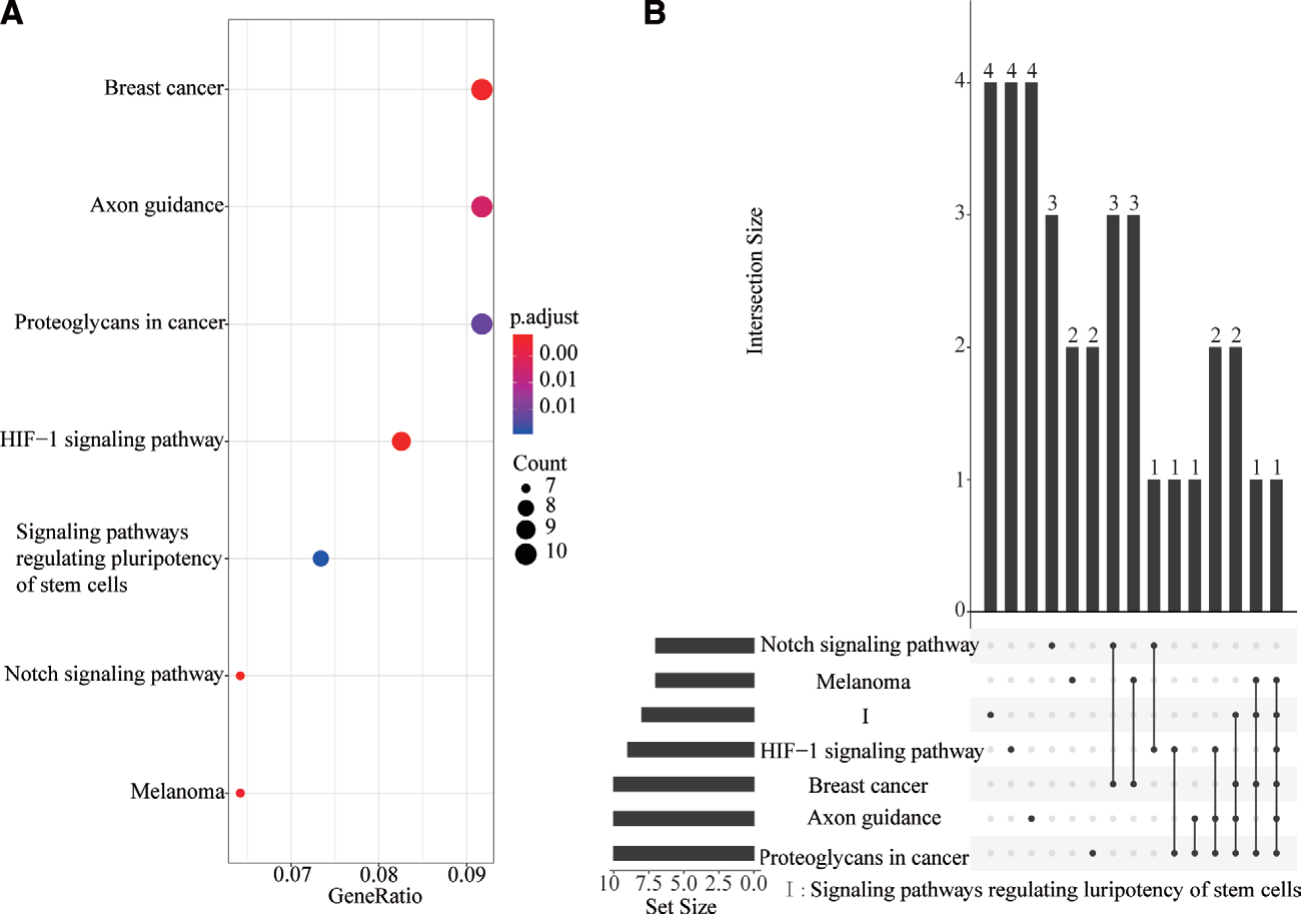
KEGG analysis of mRNA in the ceRNA regulatory network (The URL of KEGG: www.kegg.jp/kegg/kegg1.html). (A) Dotplot of KEGG analysis. (B) Upsetplot of KEGG analysis. KEGG = Kyoto encyclopedia of genes and genomes.

**Figure 6.**
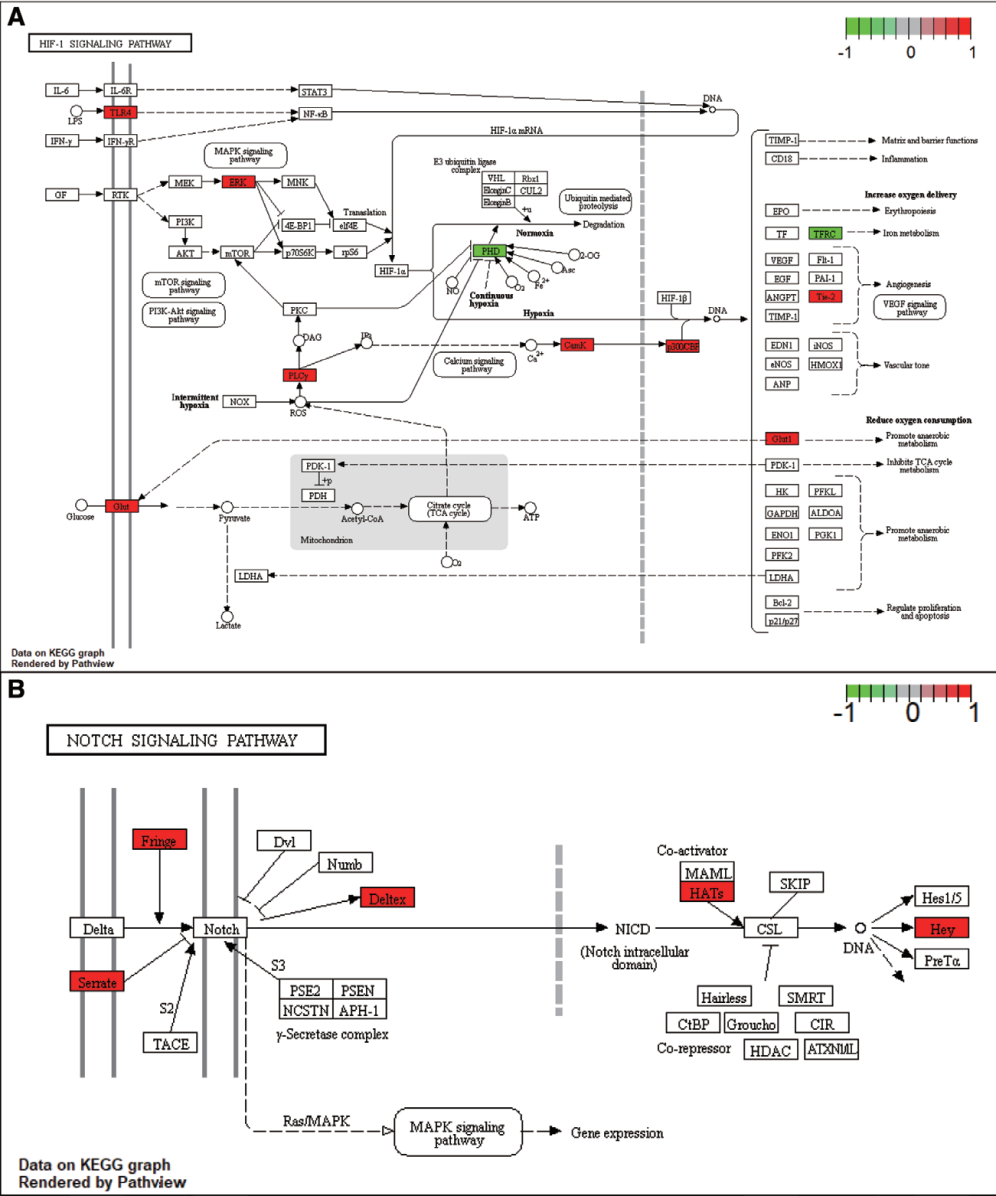
The visualization of HIF − 1 signaling pathway (A) and Notch signaling pathway (B) (The URL of KEGG: www.kegg.jp/kegg/kegg1.html). Red indicates that the relative expression level of the gene was increased, and blue indicates that the relative expression level of the gene was decreased. The darker the color, the greater the difference fold. HIF = hypoxia-inducible factors, KEGG = Kyoto encyclopedia of genes and genomes.

KEGG analysis of 274 FDEmRNA was used to determine the potential function of circRNA. The signal pathways related to circRNA functions included HIF-1 signaling pathway, Notch signaling, signaling pathways regulating pluripotency of stem cells, axon guidance, breast cancer, melanoma and proteoglycans in cancer (Fig. 5A). 274 FDE mRNAs cross different signaling pathways (Fig. 5B). For example, multiple genes in the HIF-1 signaling pathway are related to Notch signaling, proteoglycans in cancer, axon guidance, breast cancer, signaling pathways regulating pluripotency of stem cells and melanoma. HIF-1 signaling and Notch signaling pathway are reported to play important roles in the occurrence and development of IH. Therefore, we mapped the differential expression of related genes in FDEmRNA to HIF-1 and Notch signaling pathways. Multiple genes in the HIF-1 signaling pathway are activated, including TLR4, ERK, GLUT1, and Tie-2, to promote anaerobic metabolism and blood vessel growth (Fig. 6A). The same multiple genes in Notch signaling are activated, such as Fringe, Deltex, HATs, and Hey, to regulate transformation and growth of vascular endothelial cells (Fig. 6B).

### 3.5. Screening of hub genes

**Figure 7.**
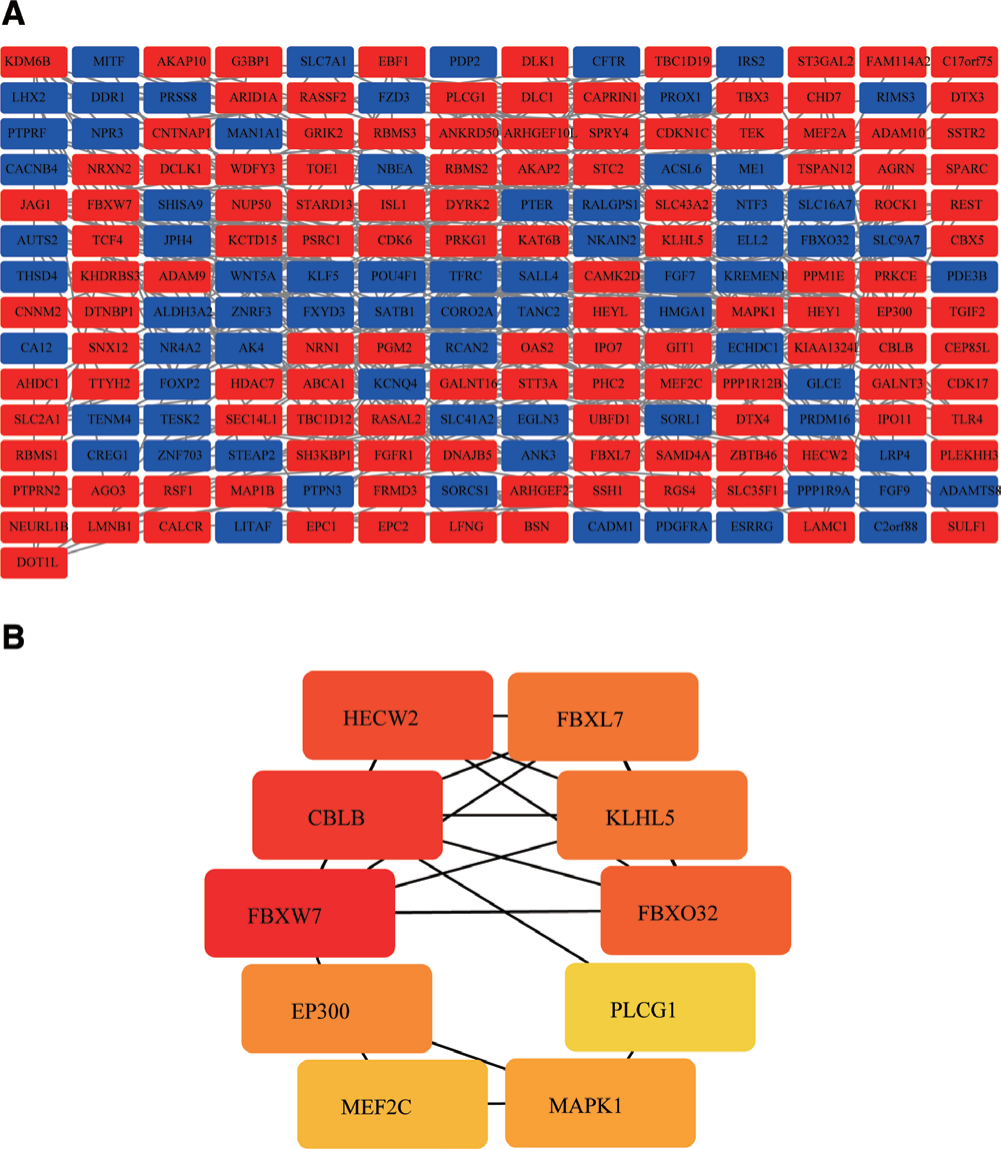
Visual analysis of the PPI network. (A) the PPI network diagram of 274 differential mRNAs in the ceRNA regulatory network; (B) the network diagram of the top 10 hub genes. PPI = protein-protein interaction.

STRING (http://string-db.org), Cytoscape and its plugin (cytoHubba) were used to identify hub genes in the ceRNA network. Figure 7A shows a protein interaction network of 274 FDEmRNAs. The network consists of 274 nodes, 351 edges. The average node degree of the network is 2.56, which represents a number of how many interactions that a protein has on the average in the network. The average local clustering coefficient is 0.392, which is a measure of how connected the nodes in the network are. The expected number of edges was 238, which represents a number of how many edges is to be expected if the nodes were to be selected at random. The *P* value of PPI enrichment was 3.79e-12 and indicated that the nodes are not random and that the observed number of edges is significant. Based on maximum clique centrality ranking by cytoHubba, the top 10 pivot genes were FBXW7, CBLB, HECW2, FBXO32, FBXL7, KLHL5, EP300, MAPK1, MEF2C, and PLCG1 (Fig. 7B).

## 4. Discussion

Infant hemangioma is the most common tumor in infants. This benign hemangioma is caused by abnormal proliferation of endothelial cells and pericytes. IH exhibits a variety of clinical features and often results in varied clinical outcomes. Most children with IH do not have life-threatening and IH regresses spontaneously. However, approximately 10% of IHs are destructive, disfiguring and even vision- or life-threatening.^[24]^ Angiogenesis and vasculogenesis are the 2 main pathogenic mechanisms of hemangioma neovascularization. These processes are related to changes in several important cell signaling pathways, including VEGF/VEGFR, Notch, β-adrenaline, Tie2/angiopoietins, PI3K/AKT/mTOR, HIF-α-mediated PDGF/ PDGF-R -β pathway.^[24]^ However, the exact mechanism of IH pathogenesis is unclear. Better understanding of the molecular pathways involved in IH pathogenesis will play an important role in guiding the development of effective and rationally designed treatments. Future research will not only provide a pharmacological basis for IH treatment, but also lay the foundation for further research on other potential anti-hemangioma drugs.

CircRNAs are stable, non-coding RNAs, whose functions in mammals are unclear.^[25,26]^ Recent studies show that circRNAs contain multiple microRNA responding elements that bind to miRNAs (“miRNA sponges”), causing release of respective downstream target mRNAs after reduced miRNAs levels in the cytosol.^[27–29]^ However, the exact role of circRNA in IH is unknown.

Here, we downloaded GSE127487, GSE69136 and GSE98795 datasets from GEO and constructed a ceRNA network based on 5403 circRNA-miRNA-mRNA axes. The network includes 108 circRNA, 7 miRNA and 274 mRNA. Our data show that some circRNAs in the ceRNA network may have important biological functions. Hsa_circ_0000092 (hsa_circRNA_001915) has been shown to promote hepatocellular carcinoma development by combining with miRNA-338-3p and upregulating HN1 expression.^[30]^ Hsa_circ_0003570 (hsa_circRNA_100709) is downregulated in liver cancer tissues and liver cancer cell lines, and is associated with liver cancer clinical features.^[31]^ Hsa_circ_0000479 (hsa_circRNA_000479) is significantly elevated in systemic lupus erythematosus and is a potential diagnostic marker.^[32]^ Hsa_circ_0043278 (hsa_circRNA_102049) promotes osteosarcoma through the miR-203a-3p/CREB3 axis, which increases tumor proliferation and invasion and inhibits apoptosis.^[33]^ Hsa_circ_0043278 also promotes proliferation, invasion and migration of non-small cell lung cancer cells and increases the expression of ROCK1, CDKN1B, and AKT3 by directly inhibiting miR-520f.^[34]^

Mounting evidence shows that aberrant circRNA expression correlates with disease pathogenesis and prognosis, suggesting that circRNA has a variety of complex functions. To investigate the mechanism of circRNA action in the ceRNA network we constructed, we did KEGG analysis of the mRNA in the ceRNA axis. This analysis found HIF-1 and Notch pathways to be enriched, and some key genes in the pathway activated, suggesting that circRNA in ceRNA network may function through HIF-1 and Notch signaling pathways. HIF-1 and Notch signaling are 2 important pathways in IH. HIF-1α expression is significantly higher in hemangioma endothelial cells relative to normal endothelial cells. HIF-α upregulation is the main driver of elevated VEGF in hemangioma endothelial cells and low HIF-α expression is associated with reduced proliferation of hemangioma endothelial cells.^[35]^ Propranolol, the first-line treatment for IH, inhibits endothelial cell proliferation in hemangioma via the HIF-1α-VEGF-A axis.^[36]^ HIF-1α signaling may also mediate autophagy during IH pathogenesis.^[37]^ There is a direct causal relationship between HIF-1α signaling and IH development. The Notch pathway might be a new regulator of VEGF signaling in IH. Suppressed Notch signaling increases blood vessel density on the superficial plexus and may lead to excessive neovascularization. Loss of Notch signal is also related to increased VEGFR-2 activity.^[38]^ Therefore, the ceRNA network constructed in this study may influence IH pathogenesis by regulating HIF-1 and Notch signaling.

In addition, the top 10 hub genes in the PPI network, including FBXW7, CBLB, HECW2, FBXO32, FBXL7, KLHL5, EP300, MAPK1, MEF2C, and PLCG1, are closely correlated with IH. Some studies have found that FBXW7 is a positive angiogenesis regulator and counters Notch activity in the endothelium of the vasculature.^[39]^ The degradation pathway mediated by FBXW7 negatively regulates HIF-1α during hypoxia.^[40]^ FBW7 also regulates endothelial function by targeting KLF2 for ubiquitination and degradation.^[41]^ HECW2, a novel EC ubiquitin E3 ligase, plays a key role in stabilizing the connections between endothelial cells by regulating AMOT-like 1 (AMOTL1) stability.^[42]^ The transcription coactivator p300 and CREB binding protein (CBP) combined with HIF-1α can stabilize the HIF-1α/β-HRE complex.^[43]^ The transcription factor MEF2C negatively controls oxygen-dependent endothelial cell angiogenesis.^[44]^ Among many cytoplasmic signaling proteins activated by VEGFR-2, PLCγ1 activation is considered a key modulator of angiogenesis.^[45]^ This analysis show that the hub gene in the ceRNA network has an important function in angiogenesis, and circRNA may regulate the progress of hemangioma through the hub gene. However, since these results are only based on bioinformatics models, further research on the possible role of ceRNA networks in IH is needed.

Although circRNA-miRNA-mRNA networks have been constructed in IH,^[46]^ the ceRNA network constructed is not perfect and needs further improvement. First, the article only used 2 circRNAs with obvious differences to construct the ceRNA network. We used all differences circRNAs to construct the ceRNA network. Additionally, the article only predicted miRNAs that interact with circRNA and mRNA, without analyzing miRNAs differences in IH. We used the GSE69136 dataset to identify differentially expressed miRNAs and constructed a ceRNA network. Finally, the mRNA dataset GSE78811 selected in that article when constructing the ceRNA network included fewer cases.

Of course, compared with other studies, the ceRNA network we constructed has certain limitations.^[47,48]^ First, there is great heterogeneity in this study, mainly because we used 3 datasets to screen the differential expression of mRNAs, miRNAs, and circRNAs in different IHs tissues, and the sample size of the datasets was relatively small. Second, due to the small sample size included in the study, we could not calculate the correlation between mRNAs, miRNAs and circRNAs. Third, we did not construct an online database, and could not visualize the ceRNA network. Finally, we did not use other algorithms including CERNIA or other classic online databases except CSCD, miRDB and Targetscan.

## 5. Conclusion

Although scholars have built circRNA-miRNA-mRNA networks in IH, the number and methods of research are not perfect. Here, we constructed a regulatory network consisting of 108 circRNAs, 7 miRNAs and 274 mRNAs through multiple GEO databases and performed functional enrichment analysis on the final target genes to understand their potential mechanism of action. Future studies will combine basic experiments with clinical data to further explore the function of these circRNAs and their potential as clinical biomarkers.

## Authors’ contributions

DG and GX designed the study, collected and analyzed data, drafted and revised the manuscript. HL and YL made substantial contributions to acquisition of data and analysis. All the authors read and approved the final version of the submitted article.

**Conceptualization:** Guangqi Xu.

**Data curation:** Da Gu.

**Formal analysis:** Da Gu, Yang Li.

**Funding acquisition:** Huanmin Lou.

**Investigation:** Huanmin Lou.

**Methodology:** Huanmin Lou.

**Project administration:** Yang Li.

**Resources:** Yang Li.

**Software:** Da Gu, Huanmin Lou.

**Supervision:** Yang Li.

**Validation:** Guangqi Xu.

**Visualization:** Da Gu.

**Writing – original draft:** Da Gu, Yang Li, Guangqi Xu.

**Writing – review & editing:** Guangqi Xu.

## Supplementary Material


